# Oral Microbiota and Immune System Crosstalk: A Translational Research

**DOI:** 10.3390/biology9060131

**Published:** 2020-06-16

**Authors:** Andrea Ballini, Gianna Dipalma, Ciro Gargiulo Isacco, Mariarosaria Boccellino, Marina Di Domenico, Luigi Santacroce, Kieu C.D. Nguyễn, Salvatore Scacco, Maura Calvani, Anna Boddi, Fabiana Corcioli, Lucio Quagliuolo, Stefania Cantore, Francesco Saverio Martelli, Francesco Inchingolo

**Affiliations:** 1 Department of Biosciences, Biotechnologies and Biopharmaceutics, University of Bari “Aldo Moro”, Campus Universitario “Ernesto Quagliariello”, 70125 Bari, Italy; andrea.ballini@uniba.it; 2 Department of Precision Medicine, University of Campania “Luigi Vanvitelli”, 80138 Naples, Italy; marina.didomenico@unicampania.it (M.D.D.); lucio.quagliuolo@unicampania.it (L.Q.); 3 Department of Basic Medical Sciences, Neurosciences and Sense Organs, University of Bari “Aldo Moro”, 70121 Bari, Italy; drciroisacco@gmail.com (C.G.I.); salvatore.scacco@uniba.it (S.S.); 4 Department of Interdisciplinary Medicine, University of Bari “Aldo Moro”, 70121 Bari, Italy; giannadipalma@tiscali.it (G.D.); francesco.martelli@ednmail.it (F.S.M.); francesco.inchingolo@uniba.it (F.I.); 5 Ionian Department, Microbiology and Virology Laboratory, Policlinico University Hospital, University of Bari “Aldo Moro”, 70124 Bari, Italy; luigi.santacroce@uniba.it; 6 Human Stem Cell’s HSC, Ho Chi Minh City 70000, Vietnam; kieugmv@gmail.com; 7 Division of Pediatric Oncology/Hematology, Meyer University Children’s Hospital, 50139 Florence, Italy; maura.calvani@meyer.it; 8 Biomolecular Diagnostic Srl, 50139 Florence, Italy; anna.boddi@bdmail.it (A.B.); fabiana.corcioli@biomoleculardiagnostic.com (F.C.); 9 IMI (International Microdentistry Institute), 50139 Florence, Italy

**Keywords:** microbiota, periodontal disease, immune system, lymphocytes, macrophage polarization, oral pathology, oral-systemic disease, clinical microbiology, clinical biochemistry, translational research

## Abstract

Background: Oral pathogens may exert the ability to trigger differently the activation of local macrophage immune responses, for instance *Porphyromonas gingivalis* and *Aggregatibacter actinomycetemcomitans* induce predominantly pro-inflammatory (M1-like phenotypes) responses, while oral commensal microbiota primarily elicits macrophage functions consistent with the anti-inflammatory (M2-like phenotypes). Methods: In healthy individuals vs. periodontal disease patients’ blood samples, the differentiation process from monocyte to M1 and M2 was conducted using two typical growth factors, the granulocyte/macrophage colony stimulating factor (GM-CSF) and the macrophage colony stimulating factor (M-CSF). Results: In contrast with the current literature our outcomes showed a noticeable increase of macrophage polarization from healthy individuals vs. periodontal patients. The biological and clinical significance of these data was discussed. Conclusions: Our translational findings showed a significant variance between control versus periodontal disease groups in M1 and M2 marker expression within the second group significantly lower skews differentiation of M2-like macrophages towards an M1-like phenotype. Macrophage polarization in periodontal tissue may be responsible for the development and progression of inflammation-induced periodontal tissue damage, including alveolar bone loss, and modulating macrophage function may be a potential strategy for periodontal disease management.

## 1. Introduction

Periodontal disease (PD) is a chronic inflammatory condition characterized by a progressive destructive disorder involving the periodontal tooth supporting tissues loss and that may negatively impact locally and systemically on health, due to an alteration of physiology balance in which it is possible to observe an over-expression of osteoclast leading to early alveolar resorption. Besides the presence of infection due the growth of specific microorganisms (dysbiosis), the host genetic/epigenetic predisposition seems to play a key role in the pathogenesis of the disease, as evidenced by the presence of single nucleotide (SNPs) or familial history predisposition [1]. The etiology of PD is composed of a complex scenario, the presence of a subgingival plaque biofilm concurrently existing with a weakened host immune response seems to be fundamental for the progression of gingivitis into periodontitis [1,2].

Several pathogens have been involved in the beginning of periodontitis, the great majority are able of generating the lipopolysaccharide (LPS), the main factor accountable for a chronic silent inflammatory state that in turn induces a change in tissue homeostasis [3]. Therefore, the loss of a coordinated host immune response eventually allows the progression of chronic periodontitis. The periodontal inflamed tissue is characterized by the broken equilibrium between pro and anti-inflammatory responses and by the accumulation of pro inflammatory cells and mediators such as leukocytes, macrophages, lymphocytes and cytokines deep into the periodontium [3,4]. Monocytes are immediately called in when an inflammatory episode begins, monocytes are recruited from either circulatory blood-derived monocytes or from local progenitors into the tissue. Here they start differentiating to macrophages, which adopt specific counter-measures accordingly to the needs and the degree of the damage they face to. Macrophages may exert different functions and they may recognize microbial components presented by the antigen presenting cells (APCs) triggering the production of selected cytokines such as interleukins (IL) like IL-1β and tumor necrosis factor alpha (TNFα) enhancing by this way cellular differentiation and maturation [3,4,5].

Macrophages play a key role in coordinating the entire phases of the inflammatory process from the initial inflammatory phase up-to the resolution. During the first step of the inflammatory process directed against pathogens (e.g., capsule, LPS and fimbriae) the macrophages trigger the receptor-mediated production of cytokines of epithelial cells with the release of neuropeptides for the vasodilation of local blood vessels [6]. Chemokines presence results in coordinating the first line of defense composed of the neutrophil that from the vessels migrate to the site of microbial invasion. Neutrophils are followed by the macrophages. This is the phase where the clinical signs of oral inflammation such as bleeding, swelling and redness of the gingiva are seen [5,6,7]. Macrophages and in specific M1 may chronically achieve the breakdown of alveolar bone and periodontal ligament inducing pockets and gingival recession, orchestrating the whole immune-pathogenesis of periodontal disease.

Macrophages are equipped with special receptors, the toll-like receptors (TLRs) that allow them to target and distinguish host and the invaders, which mediate the elimination of the pathogenic microbes through phagocytosis and the killing enzymatic mechanism. Due to the TLRs macrophages may also regulate apoptosis, inflammation and immune responses and, the recognition by the TLRs pathway induce the release of different cytokines from different cell types including the macrophages themselves [6,7,8].

Induction of specific macrophage functions is closely related to the surrounding environment that acts as a relevant orchestrator of macrophage functions. This phenomenon, termed polarization, results from cell/cell and cell/molecule interactions, governing macrophage functionality within the hosting tissues.

The evidences have emphasized the plasticity of macrophages and their inner ability to polarize to two different phenotypes, the M1 and M2, which are involved into different activities. The M1 cells regulate stimulatory signal pathways and are key factors in the classical activation of inflammatory responses while the M2 ones are mainly involved in the immune-modulatory response pathway [5,6,7,8]. The agonist/antagonist interaction is mainly seen in the production of pro-inflammatory cytokines, like IL-1β, interferon-γ (IFNγ), IL-1, TNFα and IL-6, linked to M1 and the expression of immune-regulatory cytokines, such as the IL-10 and transforming growth factor beta (TGF-β) that inactivates M1 that also contributes to the wound healing process linked to M2. It follows that any discrepancy between the M1/M2 cooperative mechanism is seen as a hallmark in the pathogenesis of many inflammatory derived chronic degenerative diseases, such as atherosclerosis, obesity, lung-fibrosis and systemic sclerosis. The main molecular difference between M1 and M2 resides in the amino acids chain compositions, such as ornithine and polyamines for M2, whereas NO (nitrogen oxide) release and citrulline characterize the M1 subset. The ornithine presence can promote cell proliferation and repair through polyamine and collagen synthesis, fibrosis and other tissue remodeling functions, while M1-produced NO is an important effector molecule with microbicide activity and cell proliferation inhibitory capacity [9,10,11,12].

**Figure 1:**
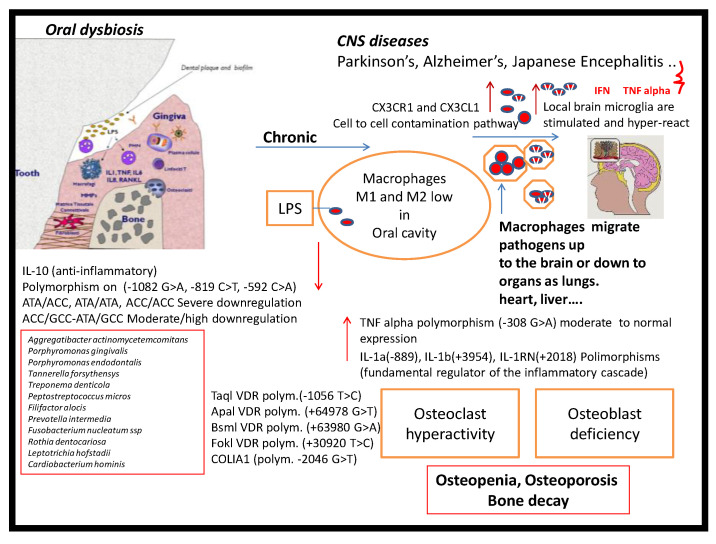
Oral dysbiosis and central nervous system (CNS) diseases: The described mechanisms may be involved in complex interactions among oral dysbiosis and CNS diseases. The central theme of CNS diseases pathogenesis is the presence of brain inflammation as illustrated by increases in pro-inflammatory cytokines, LPS and macrophages polarization. The periodontal-derived pro-inflammatory molecules and bacterial products may reach the brain via systemic circulation and/or neural pathways, contributing to brain inflammation and inflammatory vicious cycle.

Thus, macrophages with the complete set of M1 and M2 are present in periodontal lesions. The presence of macrophages within tissues is tuned due to the co-participation of colony-stimulating factor-1 or macrophage-colony-stimulating factor (CSF-1 or M-CSF), IL-34 and colony-stimulating factor-1 receptor (CSF-1R) and the matrix metallo-proteinase-9 (MMP-9) [13]. The expression of MMP-9 is required for macrophages migration at the infected location. The high presence of MMP-9 seems to be implicated in progression of periodontal disease and is indicative of M1 phenotype hyper-activity [14,15,16,17]. A critical feature of chronic periodontitis is the degradation of the collagenous structure of periodontal tissue whereas the matrix MMPs are believed to play a key role in the tissue destruction also connected to different inflammatory diseases [18,19,20,21,22,23] (Figure 1-Graphical abstract).

In this current study the main intent was to obtain in vitro both M1 and M2 from peripheral blood (PB) monocytes from two different groups of consent donors, a study group that included periodontal patients and a control group composed of healthy individuals. The differentiation process from monocyte to M1 and M2 was conducted by using two typical growth factors, the granulocyte/macrophage colony stimulating factor (GM-CSF) and the macrophage colony stimulating factor (M-CSF). A second target was to evaluate the existing differences between the macrophages obtained from the two types of groups.

## 2. Experimental Section

### 2.1. Patient Inclusion and Criteria

Totally 40 peripheral blood samples 20 PD patients (7 men and 13 women) and 20 healthy controls (5 men and 15 women) were analyzed. The mean age of participants was 50 ± 2 for men and 41 ± 5 for women. All subjects were in good general health conditions. [3].

All patients gave permission after they signed a written informed consent in accordance with the Helsinki Declaration, for the reuse of human bio-specimens in scientific research. The investigation was conducted in accordance with the current medical protocol as described by the Italian Government’s NIH legislation. The procedure followed a precise individual medical anamnesis together with the required clinical evaluations performed at the IMI (International Microdentistry Institute), Florence (Italy), in collaboration with the University of Bari “Aldo Moro” (Bari, Italy) and the University of Campania “Luigi Vanvitelli” (Naples, Italy). Procedures were conducted according to good clinical practice (GCP) and manufacture specifications. There was a prevalence of females and non-smokers (70% of the sample, 28 = females; non-smokers 85% non-smokers = 36).

Diagnosis was performed on the basis of dental clinical parameters, including periodontal probing depth (PPD), bleeding on probing (BOP), suppuration (PUS) and X-Ray images of alveolar bone condition [5]. Only patients having all PPD higher than 3 mm were included in this study. Clinical evaluation of PPD was performed using Florida Probe (www.floridaprobe.com), a computerized periodontal probing that allows tests comparable over time and independent from the operator.

Exclusion criteria included individuals with known systemic diseases, history and/or the presence of other systemic infections, antibiotic treatment preceding three months the time of current project enrolment, pregnancy or lactation.

### 2.2. Human Peripheral Blood Monocyte-Derived Macrophages

Peripheral blood sampling was performed by donors via arm veno-puncture into a vacotube of 7.5 mL with lithium heparin tubes and centrifuged with Ficoll-Paque (GE-Healthcare Sweden) in a ratio 1:3, the mononuclear cell fraction was collected and resuspended in medium RPMI-1640 (Euroclone, Milan, Italy) with 10% heat-inactivated fetal calf serum (Euroclone, Milan, Italy) and added 1 mM sodium pyruvate, 1 mM glutamine and 1% Pen/Strep (Euroclone, Milan, Italy).

Mononuclear cells (1 × 10^6^ cell/mL) were seeded in 6-well plate and incubated for 2 h at 37 °C in a 5% CO_2_ incubator. Subsequently the non-adherent cells were discarded and the adherent monocytes were cultured in RPMI-1640 with 20% heat-inactivated fetal calf serum (Euroclone, Milan, Italy) and added 1 mM sodium pyruvate, 1 mM glutamine and 1% Pen/Strep (Euroclone, Milan, Italy), supplemented with 5 ng/mL GM-CSF and 5 ng/mL M-CSF (Sigma-Aldrich, St. Louis, MO, USA) for 10 days.

Changes in cell morphology were assessed by inverted microscopy (Axio Observer Z1, Zeiss, Oberkochen, Germany). Phenotypical and functional characterization of human primary monocyte-derived macrophages (MDMs) was performed at day 7.

### 2.3. May–Grünwald Giemsa (MGG) Staining

For this technique, the slides were placed in a May–Grünwald stain solution (1:1) (Sigma-Aldrich, St. Louis, MO, USA) for 5 min and a dilute May–Grünwald solution (1:1). The slides were rinsed in deionized water and then were immerged in a freshly prepared Giemsa stain solution (dilution 1:20) for 15 min and subsequently rinsed in deionized water and dried.

### 2.4. Macrophages Immunofluorescence

The cells were grown on a glass slide (Sarsted, Leicester, UK). After a 7-days culture, the cells were fixed with 4% paraformaldehyde for 15 min, (Sigma-Aldrich, St. Louis, MO, USA) and washed with PBS 1X (Euroclone, Milan, Italy) containing either 0.1% Triton X-100, (Sigma-Aldrich, St. Louis, MO, USA) labeled with anti-CD14 polyclonal antibody at a dilution of 1:50, followed by a fluor conjugated goat anti-rabbit secondary antibody with a goat anti rabbit Alexa Fluor^®^ 488 secondary antibody to visualize the membrane (Thermo Fisher Scientific, Waltham, MA, USA). Images were assessed by inverted microscopy (Axio Observer Z1, Zeiss at resolution 100×). Nuclei were stained with a DAPI stain (Sigma-Aldrich, St. Louis, MO, USA).

### 2.5. Flow Cytometry Analysis

The differentiation to macrophages (M1 and M2) was positively confirmed by the expression of CD14, CD16, CD80, CD86, CD68, CD163 and CD206 markers as detailed below. Previous to flow cytometry cells were frozen with 90% fetal bovine serum and 10% dimethyl sulfoxide (DMSO), stored at −20 °C overnight and then at −80°. To unequivocally distinguish between macrophage subsets, the M1 or M2 were analyzed by flow cytometry analysis. On day 7 cells were harvested from cultures, cells were washed and stained with monoclonal antibodies against the following surface markers.

For the analysis of the macrophage population cells were incubated with monoclonal mouse anti-human antibodies: HLA-DR Vioblue (Miltenyi Biotec, Bergisch-Gladbach, Germany), CD16 Viobright515 FITC (Miltenyi Biotec Bergisch-Gladbach, Germany) and CD14 PE Vio770 (Miltenyi Biotec, Bergisch-Gladbach, Germany). For the analysis of the M1 population, cells were incubated with monoclonal mouse anti-human antibodies: CD80 PE, CD86 APC and CD68 APC Vio770 (Miltenyi Biotec, Bergisch-Gladbach, Germany). Instead for the analysis of the M2 population: CD163 PE Vio770 (Miltenyi Biotec, Bergisch-Gladbach, Germany) and CD206 APC Vio770 (Miltenyi Biotec, Bergisch-Gladbach, Germany).

Fluorescence-activated cell sorter (FACS) was performed on a FACSAria™ II self-service flow cytometer (Becton-Dickinson, Franklin Lakes, NJ, USA) instrument and data analyses were performed using software, and cell populations were identified using 10-color flow cytometry and sequential gating analysis.

After the exclusion of doublets and debris, macrophages were promptly identified, based on the expression of CD16, CD14 and HLA-DR. Macrophages M1 were enumerated based on their high expression of CD80, CD86 and CD68; while macrophages M2 were identified based on their expression of CD163 and CD206.

### 2.6. Statistical Analysis

Since the data presented normal distribution (Kolmogorov–Smirnov), the comparison among the obtained results by the two groups was performed using a *t*-test for unpaired samples. The correlations were evaluated via the Pearson correlation test with a 5% significance level.

The software SigmaStat 3.1 (Jandel Scientific, San Rafael, CA, USA) was used for all analyses. *p* < 0.05 was considered to indicate a statistically significant difference.

## 3. Results

### 3.1. Heterogeneity in Macrophages Morphology

Macrophages 1 and 2 were obtained from monocytes freshly isolated from whole peripheral blood from patients using the Ficoll-Paque method of isolation and cultured in the presence of GM-CSF or M-CSF for 10 days, in RPMI-1640 medium with 20% fetal calf serum respectively.

**Figure 2:**
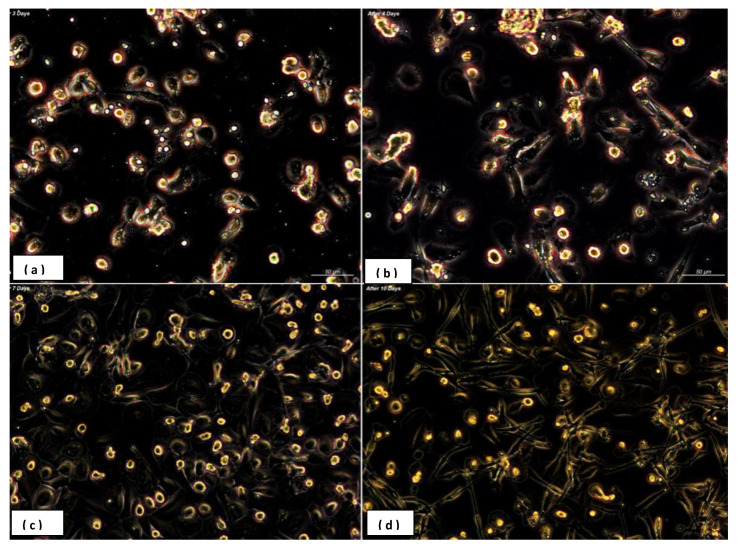
Monocyte-derived macrophages presented a unique morphology dependent on performed stimulatory effect. Granulocyte/macrophage colony stimulating factor (GM-CSF) led to a majority of elongated, fibroblast- spindle like shaped cells (**c**), and (**d**) similar to macrophages existent in lung alveoli, in contrast the presence of macrophage colony-stimulating factor (M-CSF) induced a majority of round or oval macrophages (fried eggs) like peritoneal macrophages (Axio observer Z1, Zeiss-AxioCamMR5 resolution 100×) (**a**,**b**). Human monocytes were cultured in RPMI-1640 with 20% heat-inactivated fetal calf serum supplemented with 5 ng/mL GM-CSF (granulocyte-macrophage colony-stimulating factor) and 5 ng/mL M-CSF (macrophage colony- stimulating factor). Monocytes were differentiated for 10 days in the presence of M-CSF and GM-CSF cytokines. (**a**) At day 3, (**b**) at day 4 and (**c**,**d**) at day 7 and 10. After seven days monocytes differentiated into macrophages.

The use of GM-CSF led to a majority of elongated, fibroblast-spindle like shaped cells, similar to those macrophages found in lung alveoli whereas the M-CSF induced a majority of round/oval macrophages, fried egg shape, similar to those found in peritoneal sites (Figure 2a–d). It was further observed a stronger adherence property exerted by elongated macrophages vs. round cells.

The use of GM-CSF led to a majority of elongated, fibroblast- spindle like shaped cells, similar to those macrophages found in lung alveoli whereas the M-CSF induced a majority of round/oval macrophages, fried egg shape, similar to those found in peritoneal sites (Figure 2a–d). It was further observed a stronger adherence property exerted by elongated macrophages vs. round cells.

**Figure 3:**
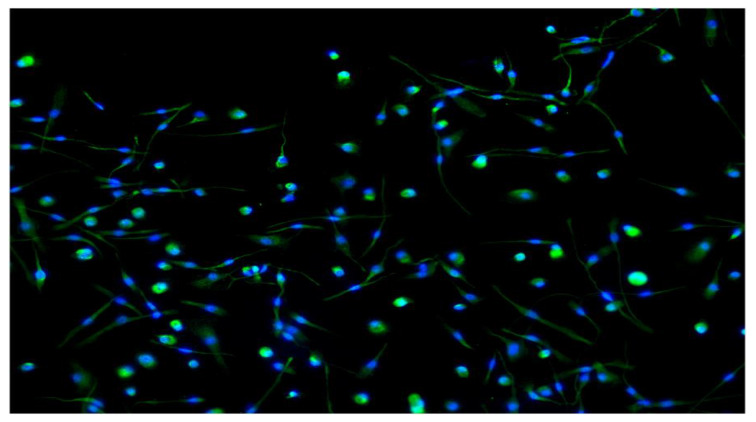
The positive expression of CD14 by immune-fluorescence analysis, which is considered to be a typical marker for the identification of macrophages. After a 7-day culture, the cells were fixed with 4% paraformaldehyde for 15 min, permeabilized with PBS (1×) containing either 0.1% Triton X-100, labeled with anti-CD14 (green stain CD14 expression with Alexa Fluor 488) polyclonal antibody at a dilution of 1:50, followed by a goat anti rabbit Alexa Fluor^®^ 488 secondary antibody to visualize the membrane. Images were assessed by inverted microscopy (Axio Observer Z1, Zeiss, resolution 100×). Nuclei were stained with DAPI. As a consequence of the growth factors used, GM-CSF led to a majority of elongated, fibroblast-spindle like shaped cells, and similar to macrophages existent in lung alveoli, in contrast the presence of M-CSF induced a majority of round or oval macrophages (fried eggs) like peritoneal macrophages.

Immunofluorescence stain analysis showed strong expression of the CD14 marker (Figure 3). CD14 is a glycosylphosphatidylinositol-anchored protein that binds LPS found on the surface of monocytes and macrophages. CD14 acts as a co-receptor for the detection of bacterial LPS. The expression of CD14 is considered to be a typical marker for the identification of macrophages [6].

### 3.2. Flow Cytometry Characterization

FACS files were analyzed using the following gating strategy: forward scatter (FSC) and side scatter (SSC) FSC-SSC identification resulted in CD14+ cells, analysis of CD80, CD86, CD68 and HLA-DR markers were performed to evaluate M1 polarization, analysis of CD163 and CD206 markers were performed to evaluate M2 polarization. Median fluorescence intensity of CD80, CD86, CD68, HLA-DR, CD163 and CD206 were conducted with FlowLogic software and summarized using both Excel and GraphPad Prism software.

**Figure 4:**
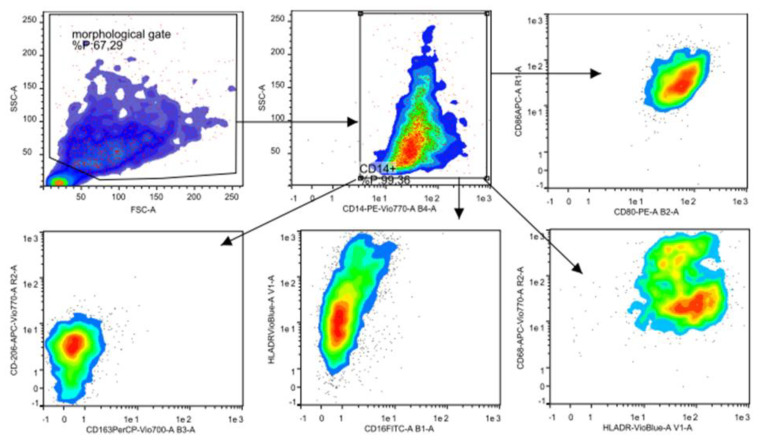
Gating strategy used for the analysis of M1 and M2 phenotype. For the analysis of human macrophage differentiation phenotype, cells were harvested and analyzed by flow cytometry. Morphological gating (top left) was used to exclude cell debris from analysis. The CD14+ monocyte differ from CD14-, in order to identify macrophages, CD14+ cells were identified (top middle). Next, CD14+ cells were analyzed for their expression of CD80 and CD86 (top right red and yellow), HLA-DR and CD68 (bottom right light green and orange), CD16 and HLA-DR (bottom middle blue) and CD163 and CD206 (bottom left green/light blue). Median fluorescence intensity of each marker was extracted by using the FlowLogic software, low-auto fluorescent cells that strongly express MHCII and CD14+ hi, high-auto fluorescent cells are marked as macrophages.

**Figure 5:**
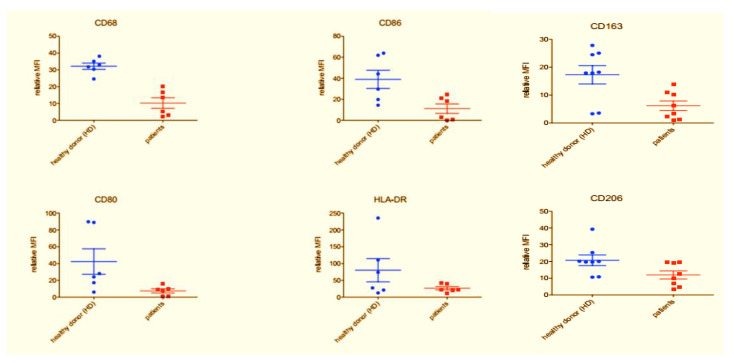
Histogram overlays obtained by using the FlowLogic software. Representative data obtained from healthy donor-derived macrophage culture (blue histograms) and patient-derived macrophage culture (red histogram). Histogram overlays were obtained by using the FlowLogic software.

For the analysis of human macrophage differentiation phenotype, cells were harvested and analyzed by flow cytometry. Morphological gating was used to exclude cell debris from analysis. In order to identify macrophages, CD14+ cells were identified (Figure 3). Next, CD14+ cells were analyzed for their expression of CD80 and CD86, HLA-DR and CD68, CD16 and HLA-DR, CD163 and CD206 (Figure 4). Median fluorescence intensity of each marker was extrapolated by using the FlowLogic software (Figure 5).

The results demonstrate that the polarization protocol induces detectable macrophages of both phenotypes M1 and M2 differentiation from human monocytes, the result is also marking a significant difference of expression of typical M1-M2 markers.

**Figure 6:**
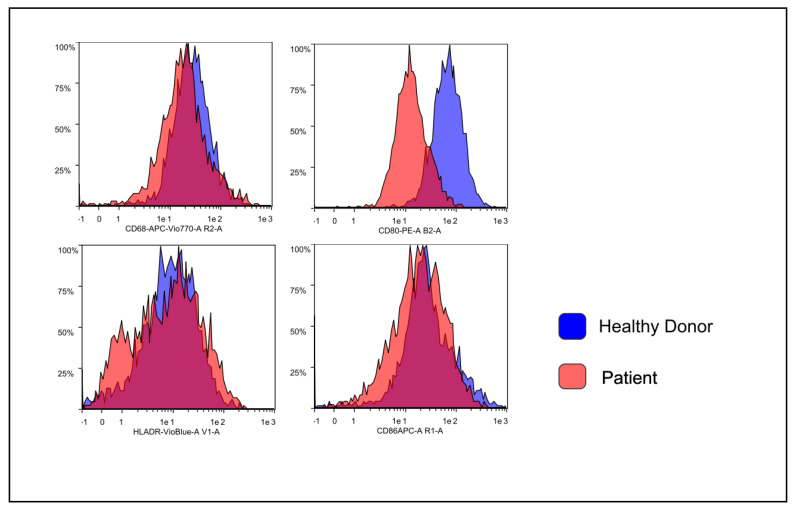
Expression of M1 markers in healthy donors and periodontal disease (PD) patients. Representative data obtained from healthy donor-derived macrophage culture (blue histograms) and patient-derived macrophage culture (red histogram). In the healthy control subjects showed a major expression of CD68 and CD86 (*p* = 0.012103) as compared to the periodontal patients, similarly for the CD80 and the HLA-DR marker but in this instance, there was not a significant difference (*p* > 0.05).

**Figure 7:**
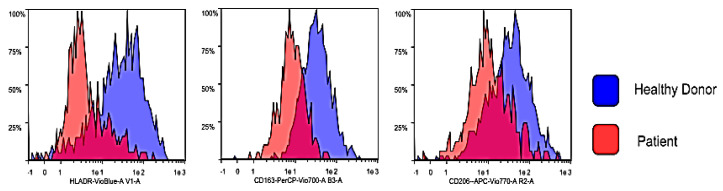
Expression of M2 markers in healthy donors and PD patients. Representative data obtained from one healthy donor-derived macrophage culture (blue histograms) and one patient-derived macrophage culture (red histogram). For the M2 markers a similar trend was observed: the healthy control subjects show a major expression of CD163 (*p* = 0.009405). For the CD206 there was a major expression, but not statistically significant (*p* = 0.063356).

The sample from patients and healthy donor showed remarkable differences. In periodontal patients, a severe decrease of the typical M1-M2 markers, as shown in the Figure 5, Figure 6 and Figure 7, was noted.

The flow cytometry analysis shows statistically significant differences between the tested groups. With regards to the M1 markers, donors defined as “healthy” showed a higher expression of CD68 (*p* = 0.016097) and CD86 (*p* = 0.012103) compared to “periodontal” samples. CD80 and HLA-DR also showed a trend similar to the previous markers however not statistically significant (*p* > 0.05). A similar behavior was observed for M2 markers. In fact, donors defined as “healthy” show a higher expression of CD163 (*p* = 0.009405) and a greater expression trend also for CD206 *(p* = 0.063356—not significant) compared to “periodontal” consider samples.

## 4. Discussion

Periodontal disease is a chronic inflammatory disease characterized by a dense inflammatory cell infiltrate including macrophages within the gingival tissue. Macrophages play a crucial role in the innate immune response and are an essential part in the pathogenesis of various inflammatory diseases. These cells play either in the initial stage of the inflammatory process or are actively involved in the immunomodulation and mediation of bone resorption and regeneration [24,25,26]. In this study, the main aims were to investigate the differentiation in vitro of human monocytes polarization towards the M1-M2 phenotypes (Figure 2 and Figure 3), and highlight the different macrophage behavior in periodontal patients and healthy individuals (study and control groups).

The GM-CSF added to fetal calf serum and culture medium showed to produce cells that recall macrophages found within lung alveoli. In contrast, macrophages derived with the use of M-CSF recalled peritoneal macrophages. The distinct macrophage subpopulations differed in their morphology and protein expression resulting in divergent functional lineages [18,19].

Though the correct sequential order between M-CSF and GM-CSF is still a matter of research, there are few significant traits that differ the two factors. GM-CS is a cytokine acting molecule, whose main activity is to stimulate stem cells to produce granulocytes like white cell subsets as neutrophils, eosinophils, basophils and monocytes. The presence of GM-CSF was based on experimental observation on lung-conditioned medium in which PB monocytes were seen to differentiate to granulocytes and macrophages [20,21].

Differently, the M-CSF is involved in different tasks and affects macrophages and monocytes by increasing both phagocytic and chemotactic activity. The M-CSF induces modulator activity on macrophages and indirectly to skeleton, skin and muscle remodeling activity due to the fact that is synthesized by a variety of different cell types, including fibroblasts, endothelial cells, bone marrow stromal cells, osteoblasts, keratinocytes, astrocytes and myoblasts. The macrophages play an important role in bone since M-CSF is produced by osteoblasts as a result of stimulation by parathyroid hormone by which it exerts paracrine effects on either osteoclasts or the M-CSF receptor.

Even if both GM-CSF/M-CSF are important in macrophage polarization, the recruitment of macrophages into a specific affected area follows different patterns from local resident macrophages. The transcriptional level leads the differences on cell response and the activation mode relies on macrophage location and on the stimulus that triggers their activation [27,28].

The majority of outcomes tend to solidly confirm an over-expression of macrophages, either M1 or M2, in the presence of PD that is usually initiated by Gram-negative anaerobic. In a recently published work of our research group, on a wider sample (96 Caucasian patients) enrolled from the same patients population included in the current study [3], we isolated, amplifying the V3 and V4 regions of 550 bp (known sequences able to identify the bacterial species present in the sample) of the bacterial 16S rRNA, pathogens traditionally associated with periodontal/peri-implant disease such as *Aggregatibacter actinomycetemcomitans*, *Porphyromonas gingivalis*, *Tannerella forsythia*, *Treponema denticola*, *Prevotella intermedia*, *Peptostreptococcus micros*, *Fusobacterium nucleatum*, *Campylobacter rectus* and *Fusobacterium nucleatum.* In addition, a bioinformatic analysis of the data obtained from the 16S rRNA V3 and V4 regions sequencing was also performed to identify and define the bacterial species that characterize the periodontal and peri-implant disease and to obtain a pattern of microbial species that identify and characterize the group of false negatives: Synergistetes, *Filifactor alocis*, *Rothia dentocariosa*, *Porphyromonas endodontalis*, *Cardiobacterium hominis*, *Leptotrichia buccalis* and *Capnocytophaga sputigena*. As well, two viruses, the Epstein–Barr Virus (EBV) and Herpes Simplex Virus 1 (HSV-1), and a pathogenic fungi (*Candida Albicans*), were included in the panel.

Moreover we also tested the following genotype frequencies (SNPs) for Hardy–Weinberg equilibrium variants, according to the literature: IL-10, TNFα (tumor necrosis factor alpha −308 G > A), IL-1α (−889); IL-1β (+3954); IL-1RN (+2018), VDRs (vitamin D receptors) Apal (+64,978 G > T), Taql (−1056 T > C), Bsml (+63,980 G > A), Fokl (+30,920 T > C) and COLIA1 (collagen type-l α; 2046 G > T), *Candida albicans*, EBV and HSV-1. The results confirmed an existing association between IL-10 gene polymorphisms and polymorphism of tumor TNFα, interleukin 1α-β-RN (IL-1α-β-RN), collagen type-l alpha (COLIA1) and vitamin D receptor (VDRs) genes in PD, which may arise under a common dysbiotic scenario following a local and systemic response. [3].

As a matter of fact, macrophages activity and their polarization towards M1 and M2 is seen as a distinctive paradigm in evaluating both activity and functionality of innate immune system responses. In addition, these types of responses to oral pathogens also showed to have systemic relation with the presence of chronic inflammatory and infectious foci far from oral location. De facto, correlations were found with diseases that eventually affect different systems like the central nervous system and guts and other mucosa [29,30,31,32].

However, in PD the real activity of macrophages and the way they exactly participate into the immune response still remain an event to be fully elucidated. The suggested assumption considers macrophages phenotypic polarization a process that may differ under the stimuli of different mediators and pathogen activity either micro-environmentally within gum and gingiva or systemically such as gut dysbiosis [33,34]. In a 2017 a study conducted by Heidari and colleagues on a small group of a group of patients who showed the complete absence of macrophage polarization [34]. The event was explained due to the interference of a specific molecule known as the macrophage inhibitory factor (MIF), a molecule highly active during the pregnancy period. The study evinced the existence of the MIF gene polymorphism may be the key condition for the total exposure to both periodontitis infection and its chronic manifestation [34,35].

A further factor that may inhibit the macrophages polarization pathway and their anti-microbial activity is the presence of a latent long-term infection. The *Brucella* and *Candida albicans* for instance, as showed by Wang and colleagues and Wagener and colleagues may inhibit the M1 and M2 formation and block macrophage antimicrobial activity [36,37]. Specifically, Wang’s study showed the inhibitory effect of macrophage exerted by *Brucella* through the LC3B-Autophagy pathway whilst Wagener’s outcomes showed the efficient blocking mechanism of *C. albicans* towards the nitric oxide production of human-monocyte-derived macrophages through to the production of host arginase-1 activity [35,36]. Intriguingly, in Wang’s study the healthy individuals showed higher expression of CD80 and CD86 M1 and CD163 and CD206 on M2 macrophages, compared to the level tested in patients affected by *Brucella* infection [35,36,37,38,39]. In this study we characterized two macrophage sub-sets the M1 and the M2, different in dimensions and shape (respectively round and spindle) by using two different growth factors: the GM-CSF and M-CSF on in vitro human monocytes cultured for 7/10 days. The differentiation has been confirmed by flow cytometry analysis with different expression of both typical M1 markers (CD80, CD86 and CD68) and M2 markers (CD163 and CD206; Figure 4 and Figure 5).

Besides, our results showed a significant variance between controls and patients groups in M1 and M2 marker expression and, according to Wang and Wagener findings [36,37], within the second group significantly lower skews differentiation of M2-like macrophages towards an M1-like phenotype have been noted and, subsequently, we can suppose that reduces phagocytosis of apoptotic cells (Figure 6 and Figure 7). These data imply that the phenotype of monocytes or macrophages is determined by their environment, such as the presence of cytokines [38,39]. The periodontitis samples obtained from the patients showed lower levels of macrophage polarized M1 and M2 compared with healthy samples collected from the control group. Our finding may reflect a perturbation in tissue homeostasis and repair in PD indicating transcriptional changes in circulating blood monocytes of affected patients. We may state, at least in vitro, that macrophages population from PD patients may have eventually lost the capacity to get to a balanced polarization of M1–M2 phenotypes as consequence of a chronic pro-inflammatory responses against oral bacterial invasion typical of PD, as finely suggested by Garalcoa-Pazmino and colleagues and Chapple and colleagues [40,41]. However, the present study provides information on the changes in monocyte/macrophages polarization of periodontitis susceptible individuals, and the results should be interpreted in this context (Figure 6 and Figure 7).

In this view it is assumed that the role of macrophages in PD is not only in accordance to the stage of the disease but is crucially related to a systemic presence of chronic aggressive pathogens and latent infection such could be in the case of Brucellosis, *C. albicans* or *Porphyromonas* [35,38].

It follows that low expression of M1 and M2 in PD patients would be the consequence of local and systemic pro-inflammatory loop lead by IL-6, IL1b and TNFα that down-regulate the macrophage polarization response, reflecting a perturbation in tissue repair in periodontitis. Likewise, considering the two common subtypes of macrophage implicated in inflammation, we determined the status of both M1 and M2 macrophages in the PD patients, the former was believed to be pro-inflammatory while the latter anti-inflammatory. As a consequence, periodontal inflammation is associated with an enhancement of both the M1 and M2 phenotypes of macrophages, in which a phenotypic switch of M2 to M1 might be a critical mechanism in mediating periodontal tissue damage, including alveolar bone loss. Even more, a weak level of local immunosuppression at the chronic inflammatory anatomical site may share a condition frequently seen in healthy tissues as an attempt to mitigate tissue destruction. However, though relatively unique and rarely detected, these events are signs specifically related to a conclusive down-regulation of macrophages, both M1 and M2, in destructive periodontitis [38,40].

The compensatory effect of well polarized M1/M2 macrophage activity is also extremely important in bone loss in PD. These patterns will better clarify.

The inner cause of the decay of bone homeostasis and regeneration in chronic periodontics patients as the balanced presence of M1 and M2 is crucial in mesenchymal stem cell derived osteoblast differentiation mechanism [38]. The role that macrophages play in bone repair and bone homeostasis mechanism whether we refer to osteocytes and osteoblasts or matrix absorber like osteoclasts is critical in such a condition.

Progression of inflammatory osteolytic condition such as those confirmed in rheumatoid arthritis (RA) and PD, is often associated by increased production of pro-inflammatory mediators and matrix-degrading enzymes where defecting M1/M2 macrophages lead to increased osteoclastic activity. Intriguingly, the macrophage migration inhibitory factor (MIF) a proinflammatory cytokine produced by T-lymphocytes and seen on neutrophils as well, plays a key role as an upstream regulator of TNFα, which has important effects in bone resorption [8].

A further aspect that could be strictly related to the M1/M2 macrophages role and their involvement in the development of different systemic degenerative diseases not only seen in bone decay but in pulmonary fibrosis (PF), neural-degenerative disease such as Alzheimer’s (AD) as well, is the chemoattractant role of MIF as ligand for CXCR4, in the recruitment of circulating osteoclast precursors (OCPs) to the bone lytic lesion. This inference has driven some authors to highpoint the role of CXR family members like the CXCR4, CCR5, CX3CR1 and CX3CL1, as a preferred way of triggering inflammatory processes by pathogens and virus for invading different tissues through M1/M2 macrophages and their cerebral counterpart, the microglial cells in the brain compartment [38]. These results may open up a future perspective that PD and CNS diseases may share common etiological pathogenic patterns through same or similar molecular/cellular pathways of which macrophages/microglial might eventually play an active part. Therefore, nearby reduced presence of macrophages that are seen in chronic periodontal patients may otherwise increase proportionally and migrate into the system scattering an inflammatory cascade. Some possible sites of inter-communication and conjunction between periphery and the brain may take place through vagal afferents, structures lacking the blood brain barrier (BBB) such as the nerve pathways from nose and eyes, through the lymphatic system, vascular endothelial cells at the and leaking BBB that allows the free passage of peripheral immune cells into the brain. The altered foreign presence into brain microenvironment hyper-stimulate local microglia, which in turn produces more pro-inflammatory cytokines. Conversely, the presence of specific inflammatory mediators released from the virus-carrying macrophages may indeed explain an unsolved periodontal or epithelial inflammation due to microorganisms and their endotoxin accumulation [42,43,44,45,46,47,48].

It has been reported that *P. ginvialis* was detected in Alzheimer’s patients. Oral *P. gingivalis* infection in mice resulted in brain colonization and increased production of Aβ1-42, a component of amyloid plaques [38]. The results of this report argue that brain infection with *P. gingivalis* is not a result of poor dental care following the onset of dementia or a consequence of late-stage disease, but is an early event that can explain the pathology found in middle-aged individuals before cognitive decline.

We can speculate from our findings that macrophages entering the CNS, may perform as the “Troy horse” effect carrying pathogens from oral affected areas that eventually led to acute and long-term microglia-mediated inflammation able to end up into a variety of CNS diseases like Alzheimer’s, Parkinson’s or dementia.

Nevertheless, the grade of reduced macrophage bactericidal immune responses could be seen as significant trait of immune infected macrophages/microglial either as hosting viruses or in chronic inflammatory condition as in periodontitis patients as reported in this study and previously confirmed by both Garalcoa-Pazmino’s and Chapple’s groups [40,41].

## 5. Conclusions

Following induction in vitro with specific stimuli the general considerations that can be drawn from these outcomes indicated in affected PD individuals a “systemic” effect defined by a general inability of isolated macrophages from peripheral blood to fully polarize into M1 or M2 phenotypes. This event may also explain the lack of polarized macrophages in vivo we are well aware that further tests are needed to confirm a differential macrophage functionality and specific cytokine secretion based on type and time of PD infection. Therefore, due to the critical role of both M1/M2 macrophages in several auto-immune diseases we also speculated that this event may also explain how CNS diseases and PD may eventually share the same or similar hyper-inflammatory macrophages/microglial pathological pathways responsible of the destructive mechanism of both cells and tissues.

In this study, based on the lower presence of both M1/M2 macrophages in PD patients compared to healthy individuals we speculated that this event could be linked to a possible common pathological scenario in which CNS diseases and periodontitis may eventually share the same or similar hyper-inflammatory macrophages/microglial pathological pathway that trigger the chronic inflammatory destructive mechanisms of both host periodontal and neural tissues.

Macrophage polarization in periodontal tissue may be responsible for the development and progression of inflammation-induced periodontal tissue damage, including alveolar bone loss, and modulating macrophage function may be a potential strategy for periodontal disease management. To conclude, more rigorous analysis to better evaluate the role of macrophages on periodontal diseases, bone and soft tissue homeostasis will certainly contribute for clearer insight of the underlining immune responses.

Therefore, we are firmly convinced that these results would open up a new methodology on periodontitis in which medical dentistry, dental surgeons and immunologists and neuro-scientists would benefit from the help of the immune-regenerative approach.
